# Factors Associated With Self-reported Use of Web and Mobile Health Apps Among US Military Veterans: Cross-sectional Survey

**DOI:** 10.2196/41767

**Published:** 2022-12-30

**Authors:** Timothy P Hogan, Bella Etingen, Jessica M Lipschitz, Stephanie L Shimada, Nicholas McMahon, Derek Bolivar, Felicia R Bixler, Dawn Irvin, Rachel Wacks, Sarah Cutrona, Kathleen L Frisbee, Bridget M Smith

**Affiliations:** 1 eHealth Partnered Evaluation Initiative Veterans Affairs Bedford Healthcare System Bedford, MA United States; 2 Center for Healthcare Organization and Implementation Research Veterans Affairs Bedford Healthcare System Bedford, MA United States; 3 Peter O'Donnell Jr School of Public Health University of Texas Southwestern Medical Center Dallas, TX United States; 4 Center of Innovation for Complex Chronic Healthcare Hines Veterans Affairs Hospital Hines, IL United States; 5 Department of Psychiatry Brigham and Women’s Hospital Boston, MA United States; 6 Harvard Medical School Boston, MA United States; 7 Department of Health Law, Policy, and Management Boston University School of Public Health Boston, MA United States; 8 Division of Health Informatics and Implementation Science, Department of Population and Quantitative Health Sciences University of Massachusetts Chan Medical School Worcester, MA United States; 9 Office of Connected Care, Veterans Health Administration US Department of Veterans Affairs Washington, DC United States; 10 Northwestern University Feinberg School of Medicine Chicago, IL United States

**Keywords:** mobile health apps, patient engagement, consumer health informatics, provider encouragement, veterans

## Abstract

**Background:**

Despite their prevalence and reported patient interest in their use, uptake of health-related apps is limited. The Veterans Health Administration (VHA) has developed a variety of apps to support veterans; however, uptake remains low nationally.

**Objective:**

We examined the prevalence of VHA health-related app use and how veterans learned about these apps in order to identify factors associated with their use.

**Methods:**

As part of a VHA quality improvement initiative, we recruited a national cohort of veterans to obtain feedback on their use of technology for health and collected data from them via a cross-sectional survey. The survey data were supplemented with VHA administrative data. We used descriptive statistics to examine demographic and health characteristics, health-related technology use, and how veterans learned about apps. We assessed factors associated with app use using bivariate analyses and multiple logistic regression models.

**Results:**

We had complete data on 1259 veterans. A majority of the sample was male (1069/1259, 84.9%), aged older than 65 years (740/1259, 58.8%), White (1086/1259, 86.3%), and non-Hispanic (1218/1259, 96.7%). Most respondents (1125/1259, 89.4%) reported being very comfortable and confident using computers, over half (675/1259, 53.6%) reported being an early adopter of technology, and almost half (595/1259, 47.3%) reported having used a VHA health-related app. Just over one-third (435/1259, 34.6%) reported that their VHA care team members encouraged them to use health-related apps. Respondents reported learning about available VHA health-related apps by reading about them on the VHA’s patient portal (468/1259, 37.2%), being told about them by their VHA health care team (316/1259, 25.1%), and reading about them on the VHA’s website (139/1259, 11%). Veterans who self-reported having used VHA health-related apps were more likely to receive care at the VHA (OR [odds ratio] 1.3, 95% CI 1.0-1.7), be in worse health (as assessed by Hierarchical Condition Community score; OR 1.1, 95% CI 1.0-1.2), report owning a desktop or laptop computer (OR 1.8, 95% CI 1.1-3.1), have posttraumatic stress disorder (OR 1.4, 95% CI 1.1-1.9), and report having VHA health care team members encourage them to use the apps (OR 2.7, 95% CI 2.1-3.4).

**Conclusions:**

We found strong associations between self-reported use by veterans of VHA health-related apps and multiple variables in our survey. The strongest association was observed between a veteran self-reporting app use and having received encouragement from their VHA health care team to use the apps. Veterans who reported receiving encouragement from their VHA care team members had nearly 3 times higher odds of using VHA apps than veterans who did not report receiving such encouragement. Our results add to growing evidence suggesting that endorsement of apps by a health care system or health care team can positively impact patient uptake and use.

## Introduction

There is an expanding number of apps available to help patients manage specific health conditions and promote overall well-being [1]. Evidence suggests use of health-related apps is associated with improved management of chronic health conditions [2] and mental health disorders [3,4], desirable health behavior change [5], and better medication adherence [6] and perceptions of health [7]. Studies have also shown that individuals are interested in using health-related apps to support self-management and to improve health [8]. Despite their prevalence and reported patient interest in using them, however, uptake of such apps remains limited [7,9,10].

A variety of barriers to health-related app adoption have been identified in previous work, including concerns about privacy and security [11-14], app usability issues [12,15], and limited proficiency with technology [14]. Recent literature also indicates that lack of awareness or knowledge of health-related apps is also a common barrier to adoption [14,16,17], highlighting the need to clarify how individuals who use health-related apps learn about them. Use of health-related apps has also been associated with certain patient demographic characteristics [18] and factors including positive perceptions of usefulness, motivation to change health behaviors or pursue a health goal, the availability of data visualization within the app, and the app not having any associated costs [9,11,14,16,18]. Importantly, research has also shown that the adoption of specific health-related apps may be bolstered if the app is recommended by a source that the target user trusts and finds credible [19].

US military veterans are an ideal population in which to examine the adoption of health-related apps. In comparison to the general US adult population, veterans often face significant health-related challenges, including disproportionate rates of physical and behavioral health diagnoses [20,21], and they commonly experience multiple comorbidities that require them to have frequent interaction with the health care system [22]. Some of these health concerns can be directly related to military service and difficulties with postservice community reintegration, while others represent common comorbidities experienced by US adults (eg, diabetes, heart disease, and chronic pain). For these reasons, US military veterans, like the broader population, stand to benefit from health-related apps and the support they offer for self-management and enhanced connection with health care providers and resources.

In recent years, studies have indicated that integrating apps into care for veterans may improve outcomes [23]. The effectiveness of multiple health-related apps targeted toward veterans has been demonstrated in randomized controlled trials, including use of the Virtual Hope Box app to support coping with negative emotions among veterans who experience suicidal ideation [24] and use of the PTSD Coach app to manage posttraumatic stress disorder (PTSD) symptom severity and increase PTSD treatment seeking [25]. As part of the Veterans Health Administration (VHA)’s digital health strategy, the VHA Office of Connected Care maintains a VHA app store, which contains a variety of mobile and web-based apps intended to support veterans in the management of their health. These apps are designed to address some of the unique needs of the veteran population, promote wellness and healthy behaviors, provide condition-specific self-management support, inform clinical management, and facilitate other transactions with the health care system that may be relevant to all veterans.

Despite the evolving evidence for their effectiveness, as well as recent literature indicating that many veterans are interested in using health-related apps [26,27] and have a device with which they can access them [28], uptake of VHA apps remains low nationally, and factors associated with veteran use of such apps are not well understood. Given that veterans represent a large patient population that could substantially benefit from the use of health-related apps, the objectives of this analysis were to examine the prevalence of VHA health-related app use, determine how veterans learned about these apps, and identify factors associated with their use.

## Methods

### Design

The VHA Office of Connected Care, in cooperation with investigators from the VHA Quality Enhancement Research Initiative program, developed the Veterans Engagement with Technology Collaborative (VET-C) cohort in 2017, the purpose of which was to engage veterans in the evaluation of VHA technologies that are intended to increase access, enhance coordination, and support self-management [28]. The VET-C cohort is a quality improvement resource that includes longitudinal survey data. Veterans who are part of the VET-C cohort are invited to provide feedback on their use of technologies for health, including VHA technologies, and this feedback is used in turn to inform usability and broader uptake. We used the VET-C cohort to examine how veterans learned about VHA health-related apps and factors associated with their adoption.

### Recruitment

To be eligible to join the VET-C cohort, veterans had to be users of VHA health care services, have a mobile phone, and be active users of the secure messaging feature of the VHA’s online patient portal, My HealtheVet, a feature that is available to veterans who have a premium portal account. The secure messaging use requirement was intended as a proxy for receptivity to and use of VHA patient-facing technologies more generally. Active use of secure messaging was defined as having sent at least 5 secure messages to VHA clinical team members through the portal in the 12 months prior to cohort recruitment. Veterans who met these inclusion criteria were recruited from VHA facilities across the United States. VHA facilities were chosen as VET-C recruitment sites because they (1) had high rates of secure messaging, (2) served as field test sites for other new VHA patient-facing technologies, (3) were known to serve significant populations of women veterans and veterans from diverse ethnic and minority groups, and (4) had active research and evaluation programs.

### Procedures

Recruitment lists to support development of the VET-C cohort were created by querying data from the VHA Corporate Data Warehouse (CDW). Veterans were called once by the evaluation team, and those who answered were told about the purpose of the VET-C cohort and invited to join. Veterans who were interested then completed the cohort baseline telephone survey, and evaluation team members entered their responses directly into an online REDCap database. During 2017 and 2018, 2727 veterans from 14 VHA facilities joined the VET-C cohort and completed the baseline survey. From March 2019 to March 2020, we administered a second survey to all veterans in the VET-C cohort who completed the baseline survey. This follow-up survey was completed by 1418 veterans in the cohort. Both our baseline and second surveys included validated question items, questions used in other studies, and new question items developed specifically for these surveys. They were developed in close consultation with leadership from the VHA Office of Connected Care, which is responsible for the health care system’s digital health strategy. After we excluded veterans for whom there were missing data for the variables used in the analyses, we included a total of 1259 veterans in the current analyses.

### Measures

For this paper, constructs of interest from the surveys included veteran demographics, health and health care use variables, technology ownership and use, VHA care team member encouragement to use VHA apps, and use of VHA health-related apps.

We asked all participants to respond to demographic questions on age, gender, race, ethnicity, relationship status, and education; report their perceived health status [29]; report where they normally received their medical care (the response options were “mostly at the VHA,” “mostly outside the VHA,” “about half in the VHA, half outside the VHA,” and “nowhere”); and indicate the amount of time it typically took them to travel to their VHA primary care doctor from their home. We also asked participants about their technology ownership (ie, whether they owned a desktop computer, tablet computer, or mobile phone), whether they considered themselves to be early adopters of technology (ie, whether they liked to be among the first to get a new device, tech gadget, or app when it comes out), and how comfortable or confident they felt using computers (responses ranged from 0, “not at all,” to 5, “very”) [30].

Additionally, we asked participants to report whether they used VHA health-related apps and how they learned about the VHA health-related apps that are available. We also asked participants to report the perceived extent to which their VHA care team members encouraged them to use health-related apps (responses ranged from 1, “strongly disagree,” to 7, “strongly agree”).

We calculated the participants’ prior-year comorbidity index as the Hierarchical Condition Community (HCC) score based on information in the VHA CDW [31,32]. The CDW was also used to identify diagnosed health conditions in the prior 5 years among our sample, and to fill in missing demographic data (eg, for age and gender).

### Analyses

We used descriptive statistics (mean, range, and SD, or proportion, as appropriate) to characterize the demographics of the sample, as well as their reported health and health care use, technology ownership and use, VHA health-related app use, how they learned about VHA health-related apps, and their perceptions of VHA care team member encouragement to use health-related apps. We used bivariate analyses (the chi-square test and the *t* test) to examine differences among veterans who reported using (vs not using) VHA health-related apps. We then assessed factors associated with VHA health-related app use using unadjusted and adjusted multiple logistic regression models. We selected variables for inclusion in the unadjusted model based on significant bivariate associations with the outcome variables and known associations from the existing literature; we selected variables for inclusion in the adjusted model based on significant unadjusted associations at the *P*<.1 level and known associations from the existing literature. Statistical analyses were performed with Stata MP (version 14.2; StataCorp).

### Ethics Approval

This work was reviewed by the institutional review boards of the VHA Bedford Healthcare System in Bedford, Massachusetts, and the Edward Hines Jr VHA Hospital in Hines, Illinois. The study was designated as a program evaluation for quality improvement purposes, exempting it from further oversight (VHA Handbook 1058.05).

## Results

### Sample Characteristics

Descriptive statistics and results of bivariate analyses are presented in Table 1. Overall, a majority of the sample was male (1069/1259, 84.9%), aged over 65 years (740/1259, 58.8%), White (1086/1259, 86.3%), and non-Hispanic (1218/1259, 96.7%). Most (1125/1259, 89.4%) reported being very comfortable and confident using computers, and over half (675/1259, 53.6%) reported being an early adopter of technology. Almost half (595/1259, 47.3%) reported using VHA health-related apps. Just over one-third (435/1259, 34.6%) reported that their VHA care team members encouraged them to use health-related apps.

Respondents reported having learned about VHA apps through the VHA’s patient portal (468/1259, 37.2%), their VHA health care team (316/1259, 25.1%), the VHA’s government website (139/1259, 11%), veteran service organizations (102/1259, 8.1%), newsletters (66/1259, 5%), other veterans (64/1259, 5%), public app stores (64/1259, 5%), and the VHA mobile app store (58/1259, 5%). These results are presented in Table 2.

**Table 1:** Comparison of demographic characteristics of veterans who were self-reported users or nonusers of health-related apps (N=1259).

Variable			Overall	Self-reported VHA^a^ app users (595/1259; 47.3%)	Self-reported VHA app nonusers (664/1259; 52.7%)	*P* value	
**Demographics, n (%)**							
	Age older than 65 years		740 (58.8)	359 (60.3)	381 (57.4)	.29	
	Male		1069 (84.9)	505 (84.9)	564 (84.9)	.97	
	**Race**						.85
		White	1086 (86.3)	512 (86.1)	574 (86.5)		
		Black	118 (9.4)	55 (9)	63 (10)		
		Other	55 (4)	28 (5)	27 (4)		
	Hispanic ethnicity		41 (3)	24 (4)	17 (3)	.14	
	Relationship status: in a relationship^b^		906 (72)	426 (71.6)	480 (72.3)	.79	
	Education status: at least some college or vocational school^c^		1115 (88.6)	524 (88.1)	591 (89)	.60	
	Socioeconomic status^d^: “not very hard to pay for basics”		897 (71.3)	413 (69.4)	484 (72.9)	.17	
Mostly receiving medical care at the VHA, n (%)			955 (75.9)	471 (79.2)	484 (72.9)	.009	
Travel time to VHA^e^: more than 60 minutes, n (%)			198 (15.7)	82 (14)	116 (17.5)	.07	
Perceived health status (fair/poor), n (%)			421 (33.4)	208 (35)	213 (32.1)	.28	
Hierarchical Condition Community score, mean (SD)			1.6 (1.2)	1.7 (1.3)	1.5 (1.1)	.047	
**Technology use, n (%)**							
	Desktop or laptop computer		1184 (94)	571 (96)	613 (92.3)	.006	
	Tablet computer		721 (57.3)	356 (59.8)	365 (55)	.08	
	Smartphone		1123 (89.2)	541 (90.9)	582 (87.7)	.06	
	Early technology adopter		675 (53.6)	327 (55)	348 (52.4)	.37	
	Very comfortable or confident using computers		1125 (89.4)	540 (90.8)	585 (88.1)	.13	
	VHA health care team encouragement to use apps		435 (34.6)	276 (46.4)	159 (24)	<.001	
**Health conditions^f^, n (%)**							
	Hypertension		726 (57.7)	345 (58)	381 (57.4)	.83	
	Osteoarthritis		716 (56.9)	347 (58.3)	369 (55.6)	.33	
	Diabetes		522 (41.5)	243 (40.8)	279 (42)	.67	
	Depression		515 (40.9)	252 (42.4)	263 (39.6)	.32	
	Chronic kidney disease		363 (28.8)	160 (26.9)	203 (30.6)	.15	
	Ischemic heart disease		340 (27)	165 (27.7)	175 (26.4)	.58	
	Asthma		302 (24)	140 (23.5)	162 (24.4)	.72	
	Posttraumatic stress disorder		269 (21.4)	148 (24.9)	121 (18.2)	.004	
	Peripheral vascular disease		202 (16)	103 (17.3)	99 (15)	.25	
	Anxiety disorders		201 (16)	102 (17.1)	99 (15)	.28	
	Atrial fibrillation		155 (12.3)	69 (12)	86 (13)	.47	
	Heart failure		118 (9.4)	62 (10)	56 (8)	.23	
	Acute myocardial infarction		115 (9.1)	48 (8)	67 (10)	.21	
	Stroke		90 (7)	45 (8)	45 (7)	.59	
	Prostate cancer		68 (5)	35 (6)	33 (5)	.48	
	Traumatic brain injury		63 (5)	33 (6)	30 (5)	.40	
	Chronic obstructive pulmonary disease		53 (4)	25 (4)	28 (4)	.99	
	Colorectal cancer		20 (2)	9 (2)	11 (2)	.84	
	Lung cancer		17 (1)	7 (1)	10 (2)	.61	

^a^VHA: Veterans Health Administration.

^b^Defined as married, in a civil union, or engaged; not being in a relationship was defined as being single, separated, divorced, or widowed.

^c^Defined as 1 to 4 years of college or vocational school or a master’s, professional, or doctoral degree.

^d^Defined by the listed response to the question “How hard is it for you (and your family) to pay for the very basics like food and heating/cooling?”

^e^Response to the question “How many minutes does it usually take you to get to your healthcare practitioners office (your VHA primary care doctor’s office)?”

^f^In the prior five years.

**Table 2:** Ways veterans reported having learned about Veterans Health Administration health-related apps. Respondents checked all options that applied to them.

Venue			Respondents (N=1259), n (%)
VHA^a^ patient portal			468 (37.2)
VHA health care team members			316 (25.1)
VHA website			139 (11)
Veteran service organizations			102 (8.1)
Newsletters			66 (5)
Other veterans			64 (5)
Public app stores			64 (5)
VHA mobile app store			58 (5)
**Other**			17 (1)
	Do not remember	12 (1)	
	At the hospital	12 (1)	
	VHA employee	7 (1)	
	Phone	6 (1)	
	Television	2 (0.2)	

^a^VHA: Veterans Health Administration.

### Bivariate Comparisons of VHA Health-Related App Users and Nonusers

Bivariate analyses comparing respondents who reported using (vs not using) VHA health-related apps revealed that the former included greater proportions of veterans with PTSD (148/595, 24.9% vs 121/664, 18.2%; *P*=.004) and veterans who reported owning a desktop or laptop computer (571/595, 96% vs 613/664, 92.3%, *P*=.006), mostly receiving their medical care at the VHA (471/595, 79.2% vs. 484/664, 72.9.%; *P*=.009), and being encouraged by their VHA care team to use the apps (276/595, 46.4% vs 159/664, 24%; *P*<.001). In addition, veterans who self-reported using VHA health-related apps had a higher average HCC score than those who did not (mean HCC score 1.7 vs 1.5, respectively; *P*=.05).

### Factors Associated with VHA Health-Related App Use

Results from the unadjusted and adjusted multiple logistic regression models assessing factors associated with self-reported VHA health-related app use are presented in Table 3. These analyses indicated that veterans who reported mostly receiving care at the VHA (OR 1.3, 95% CI 1.0-1.7), were in worse health (as assessed by HCC score; OR 1.1, 95% CI 1.0-1.2), reported owning a desktop or laptop computer (OR 1.8, 95% CI 1.1-3.1), had PTSD (OR 1.4, 95% CI 1.1-1.9), and reported having VHA health care team members encourage them to use the apps (OR 2.7, 95% CI 2.1-3.4) were more likely to self-report having used VHA health-related apps.

**Table 3:** Results of multiple logistic regression analysis of factors associated with Veterans Health Administration health-related app use (N=1259). *P<.05, **P<.01, ***P<.001.

Variable		Unadjusted odds ratio (95% CI)	Adjusted odds ratio (95% CI)
Age older than 65 years (reference: age younger than 65 years)		1.1 (0.9-1.4)	N/A^a^
Male (reference: female)		1.0 (0.7-1.4)	N/A
**Race (reference: White)**			
	Black	1.0 (0.7-1.4)	N/A
	Other	1.2 (0.7-2.0)	N/A
Hispanic ethnicity (reference: non-Hispanic ethnicity)		1.6 (0.9 -3.0)	N/A
Relationship status: in a relationship^b^ (reference: not in a relationship)		0.97 (0.8-1.2)	N/A
Education status: at least some college or vocational school (reference: less than some college education)		0.9 (0.6-1.3)	N/A
Socioeconomic status: “not very hard to pay for basics” (reference: “some hardship paying for basics”)		0.8 (0.7-1.1)	N/A
Mostly receives medical care in the VHA^c^ (reference: mostly receives medical care outside the VHA)		1.4** (1.1 -1.8)	1.3* (1.0 -1.7)
Travel time to VHA: >60 minutes (reference: ≤60 minutes)		0.8 (0.6 -1.0)	0.8 (0.6-1.1)
Perceived health status fair or poor (reference: good, very good, or excellent perceived health status)		1.1 (0.9-1.4)	N/A
Hierarchical Condition Community score (continuous)		1.1* (1.0-1.2)	1.1* (1.0-1.2)
**Technology ownership (reference: does not own a device)**			
	Desktop or laptop computer	2.0* (1.2-3.3)	1.8* (1.1-3.1)
	Tablet computer	1.2 (1.0-1.5)	1.2 (0.9-1.5)
	Smartphone	1.4 (1.0-2.0)	1.2 (0.9-1.8)
Early technology adopter (reference: not an early technology adopter)		1.1 (0.9-1.4)	N/A
Very comfortable or confident using computers (reference: not very comfortable or confident using computers)		1.3 (0.9-1.9)	N/A
VHA health care team encouragement to use apps (reference: no health care team encouragement)		2.8*** (2.2-3.5)	2.7*** (2.1-3.4)
**Health conditions^d^**			
	Hypertension	1.0 (0.8-1.3)	N/A
	Osteoarthritis	1.1 (0.9-1.4)	N/A
	Diabetes	1.0 (0.8-1.2)	N/A
	Depression	1.1 (0.9-1.4)	N/A
	Chronic kidney disease	0.8 (0.7-1.1)	N/A
	Ischemic heart disease	1.1 (0.8-1.4)	N/A
	Asthma	1.0 (0.7-1.2)	N/A
	Posttraumatic stress disorder	1.5** (1.1-2.0)	1.4* (1.1-1.9)
	Peripheral vascular disease	1.2 (0.9-1.6)	N/A
	Anxiety disorders	1.2 (0.9-1.6)	N/A
	Atrial fibrillation	0.9 (0.6-1.2)	N/A
	Heart failure	1.3 (0.9-1.9)	N/A
	Acute myocardial infarction	0.8 (0.5-1.2)	N/A
	Stroke	1.1 (0.7-1.7)	N/A
	Prostate cancer	1.2 (0.7-2.0)	N/A
	Traumatic brain injury	1.2 (0.7-2.0)	N/A
	Chronic obstructive pulmonary disease	1.0 (0.6-1.7)	N/A
	Colorectal cancer	0.9 (0.4-2.2)	N/A
	Lung cancer	0.8 (0.3-2.1)	N/A

^a^N/A: not applicable.

^b^Defined as married, in a civil union, or engaged; not being in a relationship was defined as being single, separated, divorced, or widowed.

^c^VHA: Veterans Health Administration.

^d^In the prior five years.

## Discussion

### Principal Findings

Our analyses suggest that veterans had greater odds of self-reporting use of VHA health-related apps if they mostly received their health care from the VHA, were in worse health, owned a desktop or laptop computer, and had a PTSD diagnosis. Perhaps most importantly, our analyses also showed that health care team member encouragement to use the apps was strongly associated with self-reported use. Veterans who reported receiving encouragement from their VHA care team members had nearly 3 times higher odds of using VHA apps to manage their health than veterans who did not report receiving such encouragement.

This finding confirms results from previous surveys of veterans demonstrating a positive association between health care team member recommendations to use an app and veteran interest in app use [26]. This finding is also aligned with research that extends beyond the veteran population suggesting that health-related app adoption may be bolstered if it is recommended by a source that the target user trusts and finds credible [19], such as the target user’s health care providers [14,17,33]. Taken together, our findings, along with these related studies, contribute to growing evidence regarding the importance of the role health care providers need to play to achieve widespread adoption of health-related apps. Based on this evidence, we recommend that health care systems committed to increasing the use of health-related apps in their patient populations consider how best to prepare their frontline clinical staff to engage with patients about apps that may be relevant to them. Such preparation could include, but is not limited to, educating health care team members about apps that are available for patients and evidence regarding their effectiveness, creating tools (eg, prescription pads for health-related apps or reminders and decision aids in the electronic health record) that can be used to cue action and remind patients about health care team member recommendations, and training health care team members on how best to have these conversations with patients or creating pathways for them to refer patients to other local experts who can talk with them about health-related apps.

Our analyses also revealed that certain patient demographics and health conditions were positively associated with app adoption. Prior research conducted outside the veteran population has shown that app adoption is associated with specific sociodemographic characteristics, including female sex, younger age, more education, higher socioeconomic status (SES), and better health status [9,18]. Our results differ, however, in that we did not find associations based on age, gender, education, or SES, and found an opposite association between health status and app use, namely, we found that veterans who were in worse health had greater odds of using VHA health-related apps. This may be related to our findings on encouragement; veterans who are in poorer health and those who report mostly receiving their health care from the VHA might have more frequent interactions with their providers and the health care system, which might drive increased app use. Additional research on how patients can best integrate the health-related apps they adopt into their self-management practices and sustain their use over time is also needed, as are studies of how clinical team members can best integrate the data from these apps into their clinical decision-making and workflows.

In interpreting these findings, it is important to note that our sample consisted of veterans who were established health-technology users and, as such, they may have had higher levels of technology literacy. It is possible that removing technology literacy as a barrier would affect the relationship between health status and app adoption. This is particularly important because patients who have more chronic conditions may benefit more from using health-related apps and because technology literacy is a modifiable factor [34], as it can be taught. Recent systematic reviews have underscored the importance of technology literacy as a factor in the use of consumer health informatics applications [35].

Similarly, the veterans in our sample reported having learned about VHA health-related apps from a variety of sources, the most frequently reported of which were directly tied to the VHA health care system. Sources included the VHA’s online patient portal, VHA health care team members, and the VHA’s government website. Of note, the VHA understands the importance of its online patient portal in driving adoption of other VHA health-related apps, and leverages the system to promote, market, and direct veterans to these resources in an effort to increase engagement in care and self-management. In this way, use of one patient-facing technology can beget the use of others, suggesting the importance of interventions to support the use of other technologies (as reported by Grossman et al [36]) and their potential to indirectly impact further technology adoption. Lack of awareness or knowledge of health-related apps among patients has already been recognized as a barrier to their adoption [14,16,17,37]. Our findings suggest that, at least in the case of veterans, interactions with and resources from the health care system might present effective opportunities for patients to learn about health-related apps and, in turn, overcome these barriers. While the VHA has demonstrated success using its online patient portal for this purpose, we recommend that health care systems also consider the potential of other patient-facing technologies available to their patient populations as potential platforms for promoting use of other health-related apps. For example, health care systems that are already using automated text-messaging systems to reach and remind patients could consider creating specific text messages designed to market the availability of other apps.

Of note, we also found that veterans with PTSD had greater odds of self-reporting use of VHA health-related apps. Interestingly, the results of our unadjusted analyses did not suggest that there were differences in app use among veterans with other diagnosed health conditions. While our data cannot speak to the specific reasons for this finding, we suspect that it may be driven in part by the fact that the VHA has more available and established apps relevant to PTSD, as well as conditions highly comorbid with PTSD, including depression, anxiety disorders, and insomnia, and these apps may thus be promoted more frequently than others.

Relatedly, a recent systematic review of available VHA and Department of Defense mental health–related apps found that while efficacy data for many such apps were emerging, research did indicate the efficacy of the PTSD Coach and Virtual Hope Box apps [23]. In addition, the VHA has several apps available to support behavioral health treatments commonly received by veterans with PTSD (ie, CPT Coach, PE Coach, and CBTi Coach), which behavioral health care providers may be encouraging veterans to use in tandem with treatment, thus bolstering adoption. Industry data also suggests that this trend is not specific to the VHA. In general, digital health products focused on psychiatric concerns have experienced more growth over the past decade than products focused on other health concerns [38]. We recommend health care systems see use of mental health–related apps as a potential opportunity to suggest other health-related apps to patients that may be valuable for addressing their other health and well-being needs.

### Limitations

We cannot infer causal relationships from our analyses, and self-reported survey data are subject to biases. The veterans who compose the VET-C cohort were also intentionally sampled because they were users of another VHA patient-facing technology, the health care system’s patient portal. In addition to potentially being more likely to use technology, previous research has indicated that veterans who use the VHA’s portal are more educated, younger, and have higher income than the overall veteran population [39,40], which could limit the generalizability of our findings to the overall veteran population. It is important to note, however, that to ensure the privacy and security of user’s health data, many of the VHA’s mobile apps require veterans to sign in through a secure sign-in partner, the options for which include a DS Logon Level 2 (Premium), ID.me, or My HealtheVet Premium account. In this way, the VET-C cohort, which comprises veterans who have a My HealtheVet Premium account, may more broadly reflect veterans who use the VHA’s mobile health apps. In addition, we acknowledge that the health-related apps the VHA offers are evolving, and those available at the time we completed this project may have had differing levels of relevance to the needs of different segments of the veteran population. The VET-C cohort is also characterized by more homogeneity in important demographic factors, including education and SES levels, than the overall veteran population, which may further limit generalizability. The limited number of female veterans in our sample may have further curtailed our ability to detect differences associated with gender. Finally, as with any effort to collect longitudinal data, there was attrition between the administration of our baseline survey and our second-round survey, which could have introduced response bias.

### Conclusions

In this survey of veterans, we found that nearly half of respondents self-reported use of VHA health-related apps and that encouragement from a veteran’s health care team was a critical factor associated with self-reported app use. Veterans predominantly reported learning about available health-related apps through other VHA technologies or their VHA health care team members. These results add to growing evidence suggesting that endorsement of apps by a health care system or health care providers can positively impact patient uptake and use. Future work should examine approaches to supporting efforts by health care team members to engage with patients about apps that may be most beneficial to their health, as well as ways to support shared decision-making regarding which apps to use and how best to integrate them as components of care and self-management. Such approaches could be included as part of multicomponent implementation strategies and tested to determine their impacts on the adoption of health-related apps by patients.

## Abbreviations

CDW: corporate data warehouse
HCC: Hierarchical Condition Community
OR: odds ratio
PTSD: posttraumatic stress disorder
SES: socioeconomic status
VET-C: Veterans Engagement with Technology Collaborative
VHA: Veterans Health Administration
